# Noninvasive assessment of liver steatosis in children: the clinical value of controlled attenuation parameter

**DOI:** 10.1186/s12876-017-0617-6

**Published:** 2017-05-04

**Authors:** Giovanna Ferraioli, Valeria Calcaterra, Raffaella Lissandrin, Marinella Guazzotti, Laura Maiocchi, Carmine Tinelli, Annalisa De Silvestri, Corrado Regalbuto, Gloria Pelizzo, Daniela Larizza, Carlo Filice

**Affiliations:** 1 Ultrasound Unit, Department of Infectious Diseases, Fondazione IRCCS Policlinico San Matteo, University of Pavia, Viale Camillo Golgi 19, Pavia, 27100 Italy; 2 Pediatric Unit, Department of the Mother and Child Health, Fondazione IRCCS Policlinico San Matteo, University of Pavia, Pavia, Italy; 3 Pediatric Surgery Unit, Department of the Mother and Child Health, Fondazione IRCCS Policlinico San Matteo, University of Pavia, Pavia, Italy; 4 Clinical Epidemiology and Biometric Unit, Fondazione IRCCS Policlinico San Matteo, Pavia, Italy

**Keywords:** Liver steatosis, Pediatric series, NAFLD, Obesity, Controlled attenuation parameter, Ultrasound, Transient elastography

## Abstract

**Background:**

To assess the clinical validity of controlled attenuation parameter (CAP) in the diagnosis of hepatic steatosis in a series of overweight or obese children by using the imperfect gold standard methodology.

**Methods:**

Consecutive children referred to our institution for auxological evaluation or obesity or minor elective surgery were prospectively enrolled. Anthropometric and biochemical parameters were recorded. Ultrasound (US) assessment of steatosis was carried out using ultrasound systems. CAP was obtained with the FibroScan 502 Touch device (Echosens, Paris, France). Pearson’s or Spearman’s rank correlation coefficient were used to test the association between two study variables. Optimal cutoff of CAP for detecting steatosis was 249 dB/m. The diagnostic performance of dichotomized CAP, US, body mass indexes (BMI), fatty liver index (FLI) and hepatic steatosis index (HSI) was analyzed using the imperfect gold standard methodology.

**Results:**

Three hundred five pediatric patients were enrolled. The data of both US and CAP were available for 289 children. Steatosis was detected in 50/289 (17.3%) children by US and in 77/289 (26.6%) by CAP. A moderate to good correlation was detected between CAP and BMI (*r* = 0.53), FLI (*r* = 0.55) and HSI (*r* = 0.56). In obese children a moderate to good correlation between CAP and insulin levels (*r* = 0.54) and HOMA-IR (*r* = 0.54) was also found. Dichotomized CAP showed a performance of 0.70 (sensitivity, 0.72 [0.64–0.79]; specificity, 0.98 [0.97–0.98], which was better than that of US (performance, 0.37; sensitivity, 0.46 [0.42–0.50]; specificity, 0.91 [0.89–0.92]), BMI (performance, 0.22; sensitivity, 0.75 [0.73–0.77]; specificity, 0.57 [0.55–0.60]) and FLI or HSI.

**Conclusions:**

For the evaluation of liver steatosis in children CAP performs better than US, which is the most widely used imaging technique for screening patients with a suspicion of liver steatosis. A cutoff value of CAP of 249 dB/m rules in liver steatosis with a very high specificity.

## Background

Non-alcoholic fatty liver disease (NAFLD) is becoming a major health problem in children with an increasing incidence due to sedentary lifestyles and hyper-caloric diets that are the main factors of the obesity epidemics. Ten years ago, it was estimated that in Europe roughly one obese child out of three was affected by hepatic steatosis [1]. A recent review on the burden of liver disease in Europe has reported an even higher prevalence (36–44%) [2]. A study conducted in the United States has reported that children with NAFLD have an increased risk of liver-related mortality and a survival significantly shorter than that observed in the general population of the same age and sex [3]. Thus, the diagnosis and treatment of NAFLD at an early stage is of outmost importance for limiting the progression of the pathology. Liver biopsy is the reference standard for the assessment of liver steatosis, however the procedure is invasive, has several limitations including intra and inter-observer variability in specimen’s readings, and it’s unpractical for screening purpose or to follow-up patients.

Ultrasound (US) is the imaging modality most widely used for the noninvasive assessment of liver steatosis. A good diagnostic accuracy of US for the detection of moderate to severe steatosis in children has been reported, with an area under the receiver operating characteristic (AUROC) curve of 0.87 [4]. However, US has a low sensitivity for the detection of mild steatosis [5].

Controlled attenuation parameter (CAP) is a method for the non-invasive assessment of liver steatosis. It measures the attenuation of the ultrasound beam that traverses the liver tissue, which increases in fatty liver, and is obtained by analyzing the ultrasound signal acquired by the transient elastography device (Fibroscan 502 Touch, Echosens, Paris, France) [6]. It has been shown that CAP has a good accuracy for quantifying liver steatosis also in pediatric patients [7].

The main aim of this study was to assess the clinical validity of CAP in the early diagnosis of hepatic steatosis in a series of overweight or obese children by using the imperfect gold standard methodology. Secondary aim was to analyze any association of CAP with liver stiffness values and with other indices of NAFLD.

## Methods

### Design of the study and patients

This was a single center cross-sectional study. From September 2012 to May 2016, consecutive children referred to our institution for auxological evaluation or obesity by their general practitioner or primary care pediatric consultant, scheduled for abdominal US examination and accepting to undergo also to CAP and liver stiffness measurements (LSM)s, were prospectively enrolled. Children referred to the outpatient clinic of the paediatric surgery department of our institution for minor elective surgery were also enrolled as controls.

The exclusion criteria were known secondary obesity conditions, the use of any medications, and concomitant chronic or acute illnesses.

According to the body mass indexes (BMI), the subjects were divided into three groups: obese subjects (group1): BMI that exceeded the 95th percentile for the age and sex; overweight subjects (group2): BMI 75th–95th percentile; normal weight subjects (group3): BMI < 75th percentile.

The study protocol was approved by the ethics committee of the Fondazione IRCCS Policlinico San Matteo (reference number 20120020673) and it was in accordance with the Helsinki Declaration of 1975, as revised in 2008. All the participants or their responsible guardians gave their written consent after being informed about the nature of the study.

### Physical examination and biochemical parameters

The physical examination of the participants included evaluation of height, weight, body mass index (BMI), waist circumference, pubertal stage according to Marshall and Tanner, and measurement of the blood pressure.

Metabolic blood assays included fasting blood glucose, insulin, total cholesterol, high-density lipoprotein (HDL) cholesterol, triglycerides (TG), and transaminases. Insulin resistance was determined by the homeostasis model assessment for insulin resistance (HOMA-IR) using the formula: insulin resistance = (insulin × glucose)/22.5.

Abnormalities in lipid fasting levels were considered for TG values exceeding the 95th percentile and HDL cholesterol values below the fifth percentile for age and sex. Impaired insulin sensitivity was defined with HOMA-IR that exceeded the 97.5th percentile for age and sex.

Metabolic syndrome (MS) was diagnosed according to Weiss using the criteria modified from those of the National Cholesterol Education Program’s Adult Treatment Panel III (NCEP-ATPIII) and the World Health Organization [8]. Patients were classified as having MS if they met three or more of the following criteria for age and sex: BMI > 95th percentile, TG levels > 95th percentile, HDL cholesterol level < 5th percentile, systolic and/or diastolic blood pressure > 95th percentile and fasting blood glucose >100 mg/dl and/or impaired insulin sensitivity with HOMA-IR > 97.5th percentile. In the definition by Weiss, BMI was chosen as a criterion for the MS because it correlates with visceral lipid depot, blood pressure and dyslipidemia. Although waist circumference is a good predictor of visceral adiposity in children, it might not detect differences in body proportions related to puberty and therefore no normative values exist for children and adolescents. Impaired insulin sensitivity was included because impaired fasting glucose is rare in childhood. Finally, blood pressure and fasting lipid levels were compared with population norms adjusted for age and sex.

Fatty liver index (FLI) and hepatic steatosis index (HSI) were obtained using the established formulas [9, 10]. FLI is an algorithm based on BMI, waist circumference, triglycerides and gamma-glutamyl-transferase and has been developed to detect steatosis [9], whereas HSI includes alanine aminotransferase (ALT)/aspartate aminotransferase (AST) ratio, BMI, diabetes mellitus and sex, and has been developed to detect NAFLD [10].

### Ultrasound examination, controlled attenuation parameter and liver stiffness measurements

The US assessment of liver steatosis was performed using the iU22 system (Philips Medical Systems, Bothell, USA) or the HI VISION Ascendus system (Hitachi Ltd, Japan) equipped with convex multifrequency probes. The evaluation of liver steatosis was based on a series of US findings including liver echogenicity, hepatorenal echo contrast, visualization of intrahepatic vessels, and visualization of liver parenchyma and the diaphragm. Steatosis was scored as follows: absent (score 0) steatosis was defined as normal liver echotexture; mild (score 1) steatosis as slight and diffuse increase in fine parenchymal echoes with normal visualization of diaphragm and portal vein borders; moderate (score 2) steatosis as moderate and diffuse increase in fine echoes with slightly impaired visualization of portal vein borders and diaphragm; severe (score 3) steatosis as fine echoes with poor or no visualization of portal vein borders, diaphragm, and posterior portion of the right lobe [4, 11]. The operators performing the examinations had at least 5 years of experience in US studies.

CAP was obtained by using the FibroScan 502 Touch device with the 3.5 MHz M probe or 2.5 MHz XL probes. CAP is a method for noninvasively quantifying the fat in the liver. The device estimates liver stiffness in kiloPascal (kPa) and liver steatosis in decibel/meter (dB/m). The principles of CAP have been described elsewhere [6]. CAP estimates the attenuation of the ultrasound waves at the central frequency of the probe of the Fibroscan device, and is guided by vibration-controlled transient elastography, ensuring that the operator automatically obtains the attenuation value of the liver. CAP was computed only when the associated LSM was valid and using the same signals as the one used to measure liver stiffness. As reported in the literature, only LSMs with 10 validated measurements and an interquartile range/median (IQR/M) <30% for values higher than 7.1 kPa were considered reliable [12]. There are no recommendations for successful CAP measurement. Examinations with no successful measurements after 10 attempts were deemed failures. Following a fast of at least 6 h, measurements were performed with the M probe when the skin to liver capsule distance, estimated with US, was ≤ 25 mm, otherwise the XL probe was used. In subjects with a thoracic perimeter less than 75 cm CAP measurements were not performed because the CAP is not available yet on the 5 MHz S probe, which is specifically designed for the assessment of these subjects. In each patient, the LSMs and CAP measurements were performed by the same physician who had performed the US exam.

### Statistical analysis

Power considerations: The comparison between children with obesity and children non obese, was used to investigate the discriminant validity of CAP. In the hypothesis of a significant percentage of steatosis of 50 in the first group and 20 in the second [4], a sample size of 80 in each group will have more than 90% power to detect that difference using a the two-sided Z test with pooled variance with a 0.01 significance level. In view of the possibility of missing data, at least 100 patients and 100 controls should be enrolled.

Descriptive statistics were produced for demographic characteristics for this study sample of patients. The Shapiro-Wilk test was used to test the normal distribution of quantitative variables. When quantitative variables were normally distributed, the results were expressed as the mean value and standard deviation (SD), otherwise median and interquartile range (IQR; 25th–75th percentile) were reported. Qualitative variables were summarized as counts and percentages. Pearson’s correlation coefficient was used to test the association between two quantitative continuous variables; while Spearman’s rank correlation coefficient was used to test the association between two ranked variables, or one ranked variable and one quantitative continuous variable. The correlations were categorized as follows: 0.00 to 0.25 none or slight; 0.25 to 0.50 fair to moderate; 0.50 to 0.75 moderate to good; 0.75 to 1.00 almost perfect [13].

The results of blood tests were missed for about 20% of the subjects; thus in order to calculate FLI and HSI we performed a multiple imputation of missing data. Ten datasets were created fitting regression models with TG, GGT, AST, ALT, glycaemia, waist circumference as dependent and BMI, gender and age independent variables.

The diagnostic performance of CAP, BMI with AST and/or ALT abnormal, HSI and FLI on diagnosing liver steatosis compared to the US score (gold standard) was assessed using receiver operating characteristic (ROC) curves and the area under the ROC (AUROC) curve analysis.

As a subject’s true disease status is seldom known with certainty, errors made by the gold standard mean that the sensitivity and specificity calculated for the new test are biased, and do not correctly estimate the new method’s sensitivity and specificity. Therefore we fitted two-level Bayesian latent class models with discrete latent variables to estimate sensitivity, specificity, positive predictive value (PPV), and negative predictive value (NPV) of diagnostic tests (imperfect gold standard methodology), as previously reported [14]. Briefly, the observed results of the diagnostic tests are considered as a measure, prone to error, of an unobservable (latent) dichotomous variable, i.e. the true disease status of each subject.

To fit the model, the CAP was categorized as <249 dB/m and > =249 dB/m, i.e. the optimal cutoff obtained from our data (dichotomized CAP—dCAP), HSI and FLI as <0.3 and > =0.3, i.e. the value to rule out NAFLD or steatosis, respectively [9, 10]. To improve the performance of the models the BMI of patients (categorized according to age as normal vs overweight/obese and with AST and/or ALT abnormal) was added as fourth test, since it has been previously used as an indicator of steatosis [15, 16].

*P* < 0.05 was considered statistically significant. All tests were two-sided. The data analysis was performed with the STATA statistical package (release 14.0, 2015, Stata Corporation, College (Station, Texas, USA)

## Results

Overall 305 pediatric patients were enrolled of whom 199 (103 males, 96 females; mean age, 11.5 years ([SD: 2.7]; range, 4.1–17.4) were referred for auxological evaluation or obesity and 106 (64 males, 42 females; mean age, 9.9 [SD: 3.3 years] for minor elective surgery. 100/199 (50.3%) children referred for auxological evaluation or obesity were overweight (54 males, 46 females; mean age, 11.7 [SD: 2.6] years) and 99/199 (49.7%) children were obese (50 males, 49 females; mean age, 11.0 [SD: 2.9] years). The prevalence of liver steatosis estimated using US was 24.1%. 14/106 (13.2%) children referred for minor elective surgery were overweight (7 males, 7 females; mean age, 9.2 [SD: 2.5] years) and 2/106 (1.9%) children were obese (2 females; mean age, 11.5 [SD: 6.5]). The prevalence of liver steatosis estimated using US was 2.8%.

**Fig. 1:**
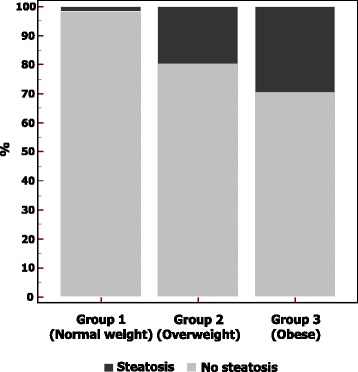
Prevalence of liver steatosis detected by US in the studied population

The children of our cohort were assigned to group1, group2, or group3 according to their BMI. The prevalence of hepatic steatosis detected by US in the three groups is shown in Fig. 1. The demographic, clinical and biochemical data of our series are reported in Table 1.

**Table 1:** Demographic, clinical and biochemical data of the studied population

Variabile	Overall *N* = 305	Group1 (Normal) *N* = 106	Group2 (Overweight) *N* = 100	Group3 (Obese) *N* = 99	*P* value #
Gender, female, *N* (%)	135 (44.8)	40 (38.5)	46 (46.9)	49 (49.4)	0.25
Age	10.9 (3.0)	10.1 (3.3)	11.8 (2.6)	11.0 (2.95)	<0.001^2^
Weight, kilograms	51.2 (19.9)	35.2 (12.2)	54.8 (13.8)	63.9 (20.4)	<0.001^*^
Height, meters	1.46 (0.16)	1.41 (0.17)	1.51 (0.14)	1.48 (0.16)	0.001^1,3^
BMI, kg/m^2^	23.1 (5.7)	17.2 (2.8)	23.6 (2.3)	28.4 (4.4)	<0.001^*^
Waist circumference, cm	76.7 (17.2)	55.3 (11.5)	79.82 (9.1)	88.48 (11.8)	<0.001^*^
Systolic blood pressure, mmHg	107.8 (10.4)	110.9 (8.54)	106.3 (10.03)	106.9 (11.5)	0.02^1^
Diastolic blood pressure, mmHg	68.7 (8.3)	70.35 (7.63)	68.15 (7.83)	68.2 (9)	0.22
Total cholesterol, mg/dl	160.3 (29.5)	156.4 (29.4)	160.6 (28.3)	162.6 (30.5)	0.44
HDL cholesterol, mg/dl	48.9 (10.8)	52.7 (12.3)	49.4 (10.6)	46.2 (9.4)	0.001^1^
AST, IU/L	22.6 (8.69)	22.7 (6.45)	21.8 (9.01)	23.2 (9.9)	0.56
ALT, IU/L	20.8 (17.2)	15.1 (5.4)	21.4 (19.6)	25.0 (19.8)	0.001^3^
GGT, IU/L	16.5 (8.5)	13.3 (5.5)	16.7 (6.2)	18.9 (11.0)	<0.001^1,3^
Fasting blood glucose, mg/dl	76.6 (10.4)	76.0 (11.9)	76.7 (10.1)	77.1 (9.4)	0.78
Fasting insulin, microIU/ml	12.7 (9.1)	9.07 (6.1)	12.69 (8.5)	13.2 (10.0)	0.34
HOMA-IR, %	2.47 (1.9)	1.83 (1.22)	2.41 (1.71)	2.6 (2.17)	0.41
Triglycerides, mg/dl	72.2 (35.1)	63.6 (29.2)	75.3 (40.2)	74.8 (32.9)	0.09
Fatty liver index	0.18 (0.21)	0.03 (0.08)	0.14 (0.12)	0.3 (0.2)	<0.001^*^
Hepatic steatosis index	0.46 (0.34)	0.13 (0.18)	0.45 (0.26)	0.7 (0.2)	<0.001^*^
CAP, decibel/meter	228.5 (50.34)	200.5 (39.36)	231.6 (51.46)	254.6 (44.4)	<0.001^*^
LSM, kiloPascal	4.70 (.93)	4.59 (.96)	4.73 (.92)	4.80 (.9)	0.30
Metabolic syndrome, *N* (%)	12 (6.09)	0 (0)	7 (9.2)	5 (6.2)	0.143

*BMI* body mass index; *HDL* high-density lipoprotein; *AST* aspartate aminotransferase; *ALT* alanine aminotransferase; *GGT* gamma glutamyltransferase; *HOMA*-*IR* homeostasis model assessment for insulin resistance; *CAP* controlled attenuation parameter; *LSM* liver stiffness measurement. Values are reported as mean and standard deviation unless otherwise specified

# P values refer to differences among three groups; ^1^ normal vs. overweight, ^2^normal vs obese, ^3^overweight vs obese: *p* < 0.05; ^*^ for all comparisons: *p* <0.01

Overall, MS occurred in five (6.2%) obese children, in seven (9.2%) overweight children and none child with normal weight (*p* = 0.12); no statistically significant difference was found between boys (9/99; 9.9%) and girls (3/98, 3.06%; *p* = 0.08).

One component of the MS was present in 46 (56.8%) obese children, 51 (67.1%) overweight children and 9 (22.5%) children with normal weight. Two components of the MS were found in 30 (37.0%) obese children, 18 (23.7%) overweight children and one (2.5%) child with normal weight.

Clinical and biochemical characteristics of the children with or without liver steatosis detected by US are reported in Table 2. Significant differences were found for the anthropometric measures, ALT (*p* < 0.001), fasting insulin (*p* = 0.01), HOMA-IR (*p* = 0.04), triglycerides (*p* = 0.004), FLI (*p* < 0.001), HSI (*p* < 0.001), CAP (*p* < 0.01), and LSM (*p* = 0.009).

**Table 2:** Clinical and biochemical characteristics of the children with and without liver steatosis detected with ultrasound

Variable	Liver steatosis ^a^		*P* value
	Yes (*N* = 50)	No (*N* = 239)	
Age, years	11.6 (2.5)	10.8 (3.04)	0.06
Weight, kg	62.2 (16.0)	48.8 (19.7)	<0.001
Height, cm	1.54 (1.4–1.6)	1.47 (1.3–1.6)	.023
BMI, kg/m^2^	26.8 (4.1)	22.2 (5.5)	<0.001
Waist circumference, cm	86.58 (11.43)	74.1 (17.4)	<0.001
Systolic blood pressure, mmHg	107.65 (11.66)	108.0 (10.1)	0.85
Diastolic blood pressure, mmHg	69.38 (7.12)	68.8 (8.5)	0.67
Total cholesterol, mg/dl	159.1 (29.13)	160.6 (29.8)	0.75
HDL cholesterol, mg/dl	46.9 (10.34)	49.28 (11.0)	0.19
AST, IU/L	24.6 (10.2)	22.18 (8.3)	0.10
ALT, IU/L	32.6 (26.3)	18.16 (13.2)	<0.001
GGT, IU/L	15.2 (6.8)	22.4 (12.3)	<0.001
Fasting blood glucose, mg/dl	76.2 (9.1)	76.8 (10.6)	0.72
Fasting insulin, microIU/ml	15.7 (10.5)	11.8 (8.4)	0.012
HOMA-IR, %	3 (2.2)	2.3 (1.8)	0.04
Triglycerides, mg/dl	86.0 (49.4)	69.1 (30.6)	0.004
Fatty liver index	0.30 (0.20)	0.15 (0.19)	<0.001
Hepatic steatosis index	0.76 (0.24)	0.39 (0.32)	<0.001
CAP, decibel/meter	280.9 (54.1)	217.4 (41.8)	<0.001
LSM, kiloPascal	5.02 (1.03)	4.65 (0.89)	0.01

*BMI* body mass index; *HDL* high-density lipoprotein; *AST* aspartate aminotransferase; *ALT* alanine aminotransferase; *GGT* gamma glutamyltransferase; *HOMA*-*IR* homeostasis model assessment for insulin resistance; *CAP*: controlled attenuation parameter; *LSM* liver stiffness measurement

^a^ Data [mean (SD)] of the 289 children for whom both US and CAP were available

### Controlled attenuation parameter

LSMs were obtained in 299/305 (98.0%) children. The six failures were due to narrow intercostal spaces. All LSMs were reliable. The M probe was used in 285/299 (95.3%) children, the XL probe in 10/299 (3.4%) children and the S probe in 4/299 (1.4%) children. In 4/299 (1.4%) the XL probe was used before the availability of CAP. Since CAP is not available yet on the S probe, only the data of 291 children examined with the M probe and XL probe were analyzed for the noninvasive assessment of liver steatosis with CAP. In two children US was not performed for technical reasons. Thus, the data of both CAP and US were available for 289 children. The mean LSM was 4.71 (SD: 0.93; range 2–7.8) kPa.

**Fig. 2:**
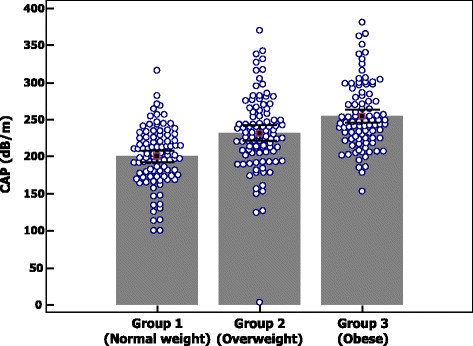
Distribution of controlled attenuation parameter (CAP) values in the three groups of children

**Fig. 3:**
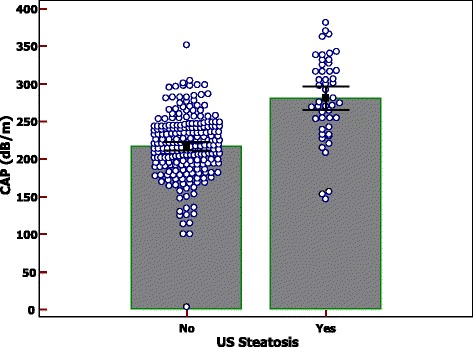
Distribution of controlled attenuation parameter (CAP) values in children without and with liver steatosis detected by ultrasound

The distribution of CAP values in the three groups of children and in children without and with liver steatosis detected by US are reported in Figs. 2 and 3. CAP values were significantly lower in children with normal weight compared to overweight and obese children (*p* < 0.001). No differences were observed in LSM between the three groups (4.59 kPa [SD: 0.96], 4.73 kPa [SD: 0.92] and 4.79 kPa [SD: 0.90]; *p* = 0.30).

Steatosis was detected by US in 50/289 (17.3%) children and by CAP in 77/289 (26.6%).

A moderate to good correlation was detected between CAP and BMI (*r* = 0.53), FLI (*r* = 0.55) and HSI (*r* = 0.56). In the obese group a moderate to good correlation between CAP and insulin levels (*r* = 0.54) and HOMA-IR (*r* = 0.54) was also found. No significant correlation was found between CAP and the other study variables.

### Diagnostic accuracy of noninvasive parameters of liver steatosis using US as the reference standard

**Fig. 4:**
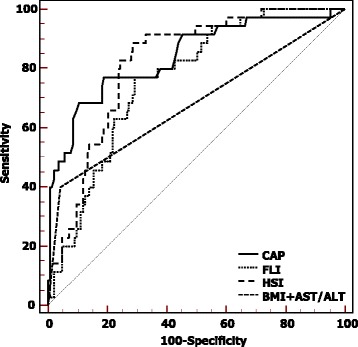
AUROCs of controlled attenuation parameter (CAP), Fatty Liver Index (FLI), Hepatic Steatosis Index (HSI), Body Mass Index (BMI) + AST and/or ALT obtained using ultrasound as the reference standard

The AUROCs of CAP, FLI, HSI, BMI + AST and/or ALT in the assessment of liver steatosis are reported in Fig. 4. The AUROC of CAP was 0.84 (95% CI:0.78–0.89, *p* < 0.001), which was higher than that of HSI (0.82; 95% CI:0.75–0.87), FLI (0.76; 95% CI:0.69–0.82), and BMI + AST and/or ALT (0.68; 95% CI:0.64–0.77).

Comparisons of AUROCs showed that CAP was significantly superior to BMI + AST and/or ALT (*p* < 0.001) whereas the differences between CAP and HSI, CAP and FLI were not statistically significant (*p* = 0.58 and *p* = 0.10, respectively).

Because several data for FLI and HSI calculation were missed, we compared FLI and HSI sensitivity/specificity calculated on complete cases and after multiple imputation of missing data. On complete cases, FLI sensitivity and specificity were 0.44 (95% CI: 0.30–0.59) and 0.83 (95% CI: 0.78–0.87), whereas HSI sensitivity and specificity were 0.93 (95% CI: 0.84–0.99) and 0.48 (95% CI: 0.42–0.54). After multiple imputation of missing data FLI and HSI sensitivity/specificity were similar to those calculated on complete cases (FLI sensitivity 0.41 [95% CI: 0.27–0.55], specificity 0.84 (95% CI: 0.79–0.89); HSI sensitivity 0.88 (95% CI: 0.79–0.98), specificity 0.51 (95% CI: 0.44–0.57).

### Performance of the noninvasive parameters of liver steatosis using the methodology without the gold standard and the Bayesian latent class model analysis

Because the models for the assessment of the performance without a gold standard require absolute independency of the diagnostic tests, we used two scenarios, one including dCAP, US, FLI, and BMI + AST and/or ALT, and the other one including the same parameters but with HSI instead of FLI. The results are reported in Table 3. In both scenarios dCAP showed the best performance with a sensitivity of 0.72 and a specificity of 0.98–1.00.

**Table 3:** Diagnostic accuracy of the noninvasive parameters of liver steatosis in the studied population using the imperfect gold standard methodology

Parameter	Sensitivity (95% CI)	Specificity (95% CI)	PPV (95% CI)	NPV (95% CI)	Performance ^a^
Analysis with the imperfect gold standard methodology: scenario 1					
dCAP	0.72 (0.64–0.79)	0.98 (0.97–0.98)	0.93 (0.90–0.96)	0.89 (0.86–0.92)	0.70
Ultrasound	0.46 (0.42–0.50)	0.91 (0.89–0.92)	0.68 (0.62–0.73)	0.80 (0.78–0.81)	0.37
Fatty Liver index	0.73 (0.72–0.74)	0.27 (0.25–0.28)	0.30 (0.29–0.31)	0.7 (0.69–0.7)	0
BMI + AST and/or ALT	0.75 (0.73–0.77)	0.57 (0.55–0.60)	0.43 (0.41–0.45)	0.84 (0.82–0.86)	0.22
Analysis with the imperfect gold standard methodology: scenario 2					
dCAP	0.72 (0.69–0.78)	1.00 (1.00–1.00)	0.99 (0.99–1)	0.89 (0.88–0.91)	0.72
Ultrasound	0.43 (0.38–0.50)	0.99 (0.99–1.00)	0.96 (0.94–0.98)	0.8 (0.78–0.82)	0.42
Hepatic Steatosis Index	0.85 (0.84–0.85)	0.33 (0.32–0.35)	0.35 (0.34–0.36)	0.83 (0.82–0.84)	0.18
BMI + AST and/or ALT	0.73 (0.69–0.77)	0.73 (0.69–0.77)	0.54 (0.49–0.58)	0.86 (0.84–0.88)	0.54

*PPV* positive predictive value; *NPV* negative predictive value; *CI* 95% Credible Interval; *dCAP* dichotomized controlled attenuation parameter; *BMI* body mass index; *AST* aspartate aminotransferase; *ALT* alanine aminotransferase

^a^ Performance = Sensitivity + Specificity - 100

## Discussion

The results of our study show that the diagnostic performance of CAP for the evaluation of liver steatosis was higher than that of US. Due to its noninvasiveness, availability, relatively low cost and repeatability because there is no exposition to ionizing radiation, US is the most widely used technique for screening patients with a suspicion of liver steatosis. Over the years, the technological advancement of the US systems has led to an increase of the sensitivity of technique, which has been reported to be 0.90 in detecting steatosis involving at least 20% of the hepatocytes [17]. However, for lower levels of fat infiltration a negative US result does not rule out the presence of liver steatosis. Especially in children, it is of outmost importance to diagnose NAFLD at an early stage because a modification of the diet and of the lifestyle can avoid the progression to end-stage liver disease, whose incidence seems higher than in adults [18, 19]. Magnetic resonance spectroscopy is the most accurate method for the detection of fat in the liver, however the technique is costly and available only in few referral centers, and often requires sedation in children. CAP is a new noninvasive method that has shown promise in the assessment of steatosis also in pediatric patients. Recently, in a study performed in a small series of children in whom liver biopsy was carried out for clinical indications, a cut point of 225 dB/m for predicting steatosis has been identified, with 0.87 sensitivity, 0.83 specificity, and AUROC 0.93 [7]. The results of our study show that a CAP cutoff value of 249 dB/m rules in liver steatosis with a very high specificity. Moreover, steatosis was detected with CAP in 27 (9.3%) children with normal US findings. This finding suggests that CAP could be a useful tool for the diagnosis of liver steatosis at an early stage.

In our series, children with liver steatosis showed LSMs significantly higher than those observed in children without steatosis by some 0.5 kPa. Although this difference was not relevant from a clinical point of view because these values were still in the normal range, we would like to underline that significant liver fibrosis was observed in one child with liver steatosis and in none child without. This finding confirms that liver steatosis is not always a benign condition, thus care should be taken to assess the disease at an early stage. On this regard, CAP seems a very useful tool.

FLI and HSI are biochemical indices of hepatic steatosis that have been derived and a currently used in the adult population, whereas data on children are scarce. In our series, similar to what observed in adults, a moderate to good correlation of CAP with these indices was detected, indicating that FLI and HSI could be used as noninvasive biomarkers of liver steatosis in the pediatric population as well. However, it has been reported that these indices have insufficient diagnostic accuracy for diagnosing or excluding NAFLD in severely obese children [20].

This study has limitations. First, we used the methodology without a gold standard since liver biopsy is not indicated for screening purpose even in a high risk population. The latent class analysis method for estimating test accuracy in the absence of a gold standard is well documented and has been applied in several studies [21]. Second, the biochemical data were not available for all children, thus we used the multiple imputation of missing data. However, the sensitivity and specificity were similar to those calculated with the complete data. Third, the children in our study were enrolled from pediatric patients referred to the hospital for auxological evaluation or obesity or elective minor surgery, therefore the results may not be applicable to the general population. Forth, since we used US as the reference standard to calculate the cutoff value of CAP for the detection of steatosis, we could have underestimated liver steatosis in our cohort. It is likely that with a lower cutoff, as the one obtained in the series of Desai et al [7] where liver biopsy was the reference standard, the performance of CAP would have been even higher than observed in our population.

## Conclusions

For the evaluation of liver steatosis in children CAP performs better than US, which is the most widely used imaging technique for screening patients with a suspicion of liver steatosis. A cutoff value of CAP of 249 dB/m rules in liver steatosis with a very high specificity.

## Abbreviations

ALT: Alanine aminotransferase
AST: Aspartate aminotransferase
AUROC: Area under the receiver operating characteristic
BMI: Body mass index
CAP: Controlled attenuation parameter
CI: Confidence interval
dB/m: Decibel/meter
dCAP: Dichotomized CAP
FLI: Fatty liver index
HDL: High-density lipoprotein
HOMA-IR: Homeostasis model assessment for insulin resistance
HSI: Hepatic steatosis index
IQR: Interquartile range
IQR/M: Interquartile range/median
kPa: kiloPascal
LSM: Liver stiffness measurements
MS: Metabolic syndrome
NAFLD: Non-alcoholic fatty liver disease
NCEP-ATP: National Cholesterol Education Program’s Adult Treatment Panel
NPV: Negative predictive value
PPV: Positive predictive value
ROC: Receiver operating characteristic
SD: Standard deviation
TG: Triglycerides
US: Ultrasound
